# Neoatherosclerosis in Very Late Stenosis of Bare Metal Stent by Optical Coherence Tomography

**DOI:** 10.1155/2016/1652065

**Published:** 2016-03-13

**Authors:** Samer Mowakeaa, Aline Iskandar, Nikolaos Kakouros

**Affiliations:** Department of Cardiology, University of Massachusetts Medical School, Worcester, MA 01655, USA

## Abstract

Bare metal stents (BMS) continue to be widely used in patients with coronary artery disease undergoing percutaneous revascularization. Progressive luminal renarrowing has been reported late after BMS implantation resulting in a significant rate of stent failure events. We present a case of very late BMS failure due to in-stent restenosis where optical coherence tomography (OCT) was used to demonstrate neoatherosclerosis as the underlying mechanism. We provide a brief review of neoatherosclerosis and showcase salient features on OCT evaluation.

## 1. Introduction

Despite the high rates of drug-eluting stent utilization in the United States [1], bare metal stents (BMS) continue to be widely used in patients with coronary artery disease undergoing percutaneous revascularization (15% of the patients in 2014) [2]. Very late stent failure, occurring >10 years after initial implantation, is not an infrequent finding with bare metal stents (BMS) [3]. Progressive luminal renarrowing beyond 4 years after BMS placement has been reported previously [3, 4]. Although multiple factors contributing to late stent failure have been proposed, de novo development of atherosclerosis within the neointima has been identified as a major cause [5, 6].

Optical coherence tomography (OCT) is an intravascular imaging modality that uses near-infrared light and provides high resolution images of the vascular wall with a detailed assessment of neointimal tissue [7]. Thus, OCT can be a valuable tool used to identify the mechanism of stent failure by demonstrating distinct tissue characteristics.

## 2. Case Report

**Figure 1:**
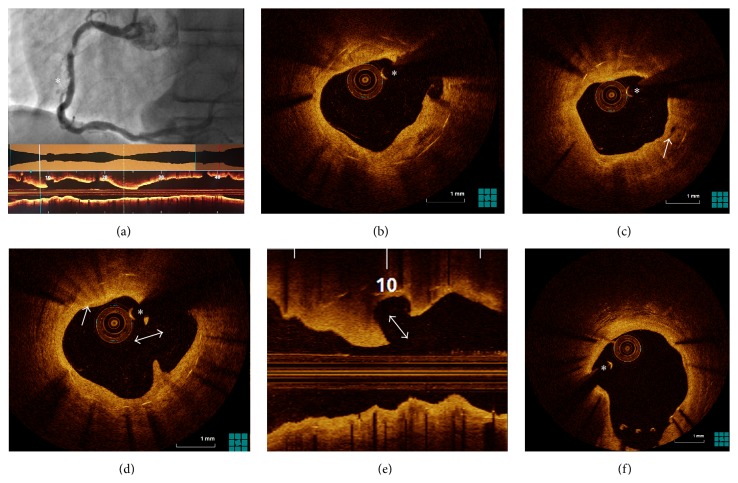
(a) Coronary angiography showing a severe stenosis within the stent as well as luminal contour suggestive of plaque rupture more distally (asterisk). An L-mode OCT confirms severe luminal stenosis. (b) OCT showing neoatherosclerosis with lipid pools and a well-defined heterogenous low signal area of fibrocalcific deposits (arrow) within the previous stent. (c) Microvessels (arrow) within fibrotic neointimal tissue. (d) Intrastent ruptured plaque with disrupted fibrous cap and evacuated core. Active macrophage infiltration is also noted with punctate, reflective, and highly attenuating regions (arrow), as is an old plaque rupture with the absence of thrombus (double arrow). (e) Longitudinal OCT view showing old ruptured plaque within the stent, with aperture facing the direction of blood flow (double arrow). (f) Incidental finding of malapposed uncovered struts at the proximal stent segment with no evidence of thrombus. The asterisk indicates the guide-wire artifact.

A 66-year-old male was admitted to our hospital with an episode of chest pain during atrial fibrillation with rapid ventricular response following a month history of progressive exertional chest pain. He had been treated 15 years previously with two overlapping Duet 3.5 × 23 mm and 3.5 × 13 mm BMS to his proximal and mid right coronary artery. Electrocardiography did not show ischemic changes at rest, but a significant rise in cardiac biomarkers was noted, consistent with a diagnosis of non-ST-elevation myocardial infarction. Coronary angiography demonstrated a critical stenosis within the prior stent in the mid-RCA (Figure 1(a)) that was occlusive to flow when crossed with a Dragonfly Duo OCT catheter. Focal predilation of the severe stenosis was performed using a Trek 3.0 × 12 mm balloon to improve distal vessel flow. Subsequent OCT confirmed severe in-stent restenosis with a percent area stenosis of 78% and minimal luminal area of 2.3 mm^2^. OCT also revealed the presence of neoatherosclerosis with fibrocalcific plaque, lipid pools, macrophage accumulation, and neoangiogenesis (Figures 1(b)–1(d)) throughout the majority of the stent's length. A site of old plaque rupture was visualized within the stented segment neoatheroma (Figure 1(e)) with no evidence of acute thrombus. An incidental finding of malapposed uncovered struts at the proximal stent segment was also seen (Figure 1(f)).

## 3. Discussion

The present study illustrates a case of very late stent failure due to severe in-stent restenosis (ISR) causing progressive exertional angina and a type 2 myocardial infarction. Intravascular imaging with the use of OCT was used to help identify the underlying mechanism of stent failure revealing distinct features consistent with the presence of neoatherosclerosis.

A series of luminal changes have been previously described following BMS implantation [4]. Emerging data suggests that neoatherosclerosis plays a major role in the pathophysiology of late ISR (beyond 4 years) [5, 6]. Neoatherosclerosis is histologically characterized by foamy macrophage clusters that accumulate forming fibroatheromas, with or without a necrotic core and/or calcification within the neointima [8]. Further infiltration of foamy macrophages within the neointima results in the formation of a thin-cap fibroatheroma, which may in turn lead to in-stent plaque rupture. In all cases, there is no communication between the in-stent lesion and the underlying native atherosclerotic plaque. As demonstrated by OCT images in our case, the ISR lesion showed similar findings (Figures 1(b)–1(e)). The exact elements leading to development of neoatherosclerosis are unknown, but it is speculated that endothelial dysfunction contributes to the process [6, 8].

OCT provides superior resolution intravascular images of the neointima. Characterization of neoatherosclerotic tissue by OCT has been described previously [8]. Foamy macrophage accumulation appears as a thin bright signal with a trailing shadow. A necrotic core is visualized as high attenuation signal poor region with poorly defined borders. It is important to recognize, however, that signal poor areas in OCT imaging are not exclusively caused by necrotic core (other causes for poor signal areas include fibrin accumulation, granulation tissue, lipid pool, and organized thrombus) [8].

Identifying the underlying process resulting in very late stent failure in our patient is the first step to better understand its development and allow for further research of targeted preventative therapies.

## 4. Conclusion

Neoatherosclerosis is an important cause of very late ISR and stent thrombosis [9]. This study showcases characteristic findings and highlights the utility of OCT in the evaluation of the coronary pathophysiology.
